# Choice of size-exclusion chromatography column affects recovery, purity, and miRNA cargo analysis of extracellular vesicles from human plasma

**DOI:** 10.20517/evcna.2024.34

**Published:** 2024-09-06

**Authors:** Jillian W.P. Bracht, Mandy Los, Edwin van der Pol, Sandra A.W.M. Verkuijlen, Monique A.J. van Eijndhoven, D. Michiel Pegtel, Rienk Nieuwland

**Affiliations:** ^1^Department of Clinical Chemistry, Laboratory of Experimental Clinical Chemistry, Amsterdam Vesicle Center, Amsterdam UMC, location University of Amsterdam, Amsterdam 1105 AZ, the Netherlands.; ^2^Cancer Centre Amsterdam, Imaging and Biomarkers, Amsterdam UMC, location University of Amsterdam, Amsterdam 1105 AZ, the Netherlands.; ^3^Amsterdam Cardiovascular Sciences, Atherosclerosis and Ischemic Syndromes, Amsterdam UMC, location University of Amsterdam, Amsterdam 1105 AZ, the Netherlands.; ^4^Department of Biomedical Engineering and Physics, Amsterdam UMC, location University of Amsterdam, Amsterdam 1105 AZ, the Netherlands.; ^5^Department of Pathology, Amsterdam UMC, location Vrije Universiteit Amsterdam, Amsterdam 1081 HV, the Netherlands.

**Keywords:** Size-exclusion chromatography, extracellular vesicles, miRNA, human plasma, lipoproteins

## Abstract

**Aim:**

The miRNA cargo of plasma extracellular vesicles (EVs) is commonly studied for its biomarker potential. However, isolation of EVs from human plasma is challenging. Although size-exclusion chromatography (SEC) is commonly used to isolate plasma EVs, SEC does not completely separate EVs from other miRNA carriers such as cells, lipoproteins, and proteins. Recently, new SEC columns were introduced, but hitherto, no systematic study was performed to compare the recovery and purity of plasma EVs using both traditional and new columns. In this study, we investigated the recovery of EVs and separation efficacy from lipoproteins and proteins using different SEC columns, and how recovery and separation affect miRNA cargo analysis.

**Methods:**

EVs were isolated from pooled (*n* = 5) platelet-depleted plasma using 10 different SEC columns. For each column, three EV-enriched fractions were pooled and concentrations of EVs, lipoproteins, proteins, and miRNAs were measured by flow cytometry, enzyme-linked immunosorbent assay (ELISA), Bradford assay, and qRT-PCR, respectively.

**Results:**

Our results show that the resin pore size affects all measured parameters: a small pore size increases recovery of EVs and quantity of miRNA, but decreases the separation efficacy compared to a large pore size. Regression analysis showed that the investigated miRNAs are more strongly associated with EVs than with lipoproteins or proteins.

**Conclusion:**

The choice of a SEC column markedly affects the recovery, separation efficacy, and miRNA cargo analysis of human plasma-derived EVs. We recommend either using SEC columns with a 70-nm pore size due to their superior EV purity or studying the effect of non-EV particles on the miRNAs of interest.

## INTRODUCTION

Extracellular vesicles (EVs) are membrane-delimited particles that are released by cells into body fluids such as blood. The EV concentration and their molecular cargo, such as microRNAs (miRNAs), are thought to reflect changes in health and disease, and therefore, EVs are thought to be a valuable source of biomarkers^[1-3]^.

Blood plasma contains about ~10^7^-10^10^ EVs per mL^[3-6]^ (diameter range: 30-1,000 nm), and is prepared by centrifugation to remove cells^[7]^. Despite centrifugation, however, the obtained plasma still contains miRNA carriers other than EVs such as platelets (2-4 µm, ~10^4^-10^7^ per mL)^[8-10]^, erythrocyte ghosts (7-8 µm, ~10^5^-10^6^ per mL)^[9,10]^, proteins and protein aggregates (1-1.5 × 10^4^ nm, ~10^16^-10^17^ per mL)^[11,12]^, and lipoproteins (5-1.2 × 10^3^ nm, ~10^15^-10^16^ per mL, depending on the prandial status)^[4,12,13]^. The presence of non-EV miRNA carriers likely interferes with the miRNA cargo analyses of EVs^[3,14-16]^, and therefore, the non-EV miRNA carriers should be separated from plasma EVs prior to downstream analyses^[17]^. Recently, we showed that platelets can be removed from plasma by filtration without affecting the concentration of EVs^[8,10]^. Nevertheless, this platelet-depleted plasma still contains lipoproteins and proteins, both of which are known carriers of miRNAs.

At present, there is not a single method available that completely separates EVs from lipoproteins and proteins. The most commonly used method to enrich EVs relative to lipoproteins and proteins is size-exclusion chromatography (SEC), which separates particles based on size^[12]^. The principle of this size-based separation method is that particles exceeding the diameter of the resin pores migrate with the fluid stream, whereas smaller particles enter the resin pores and therefore elute later than the large particles. When plasma is used as starting material, SEC2B (Sepharose 2B resin) reduces the concentration of small lipoproteins (e.g., HDL particles) and proteins by ~99%^[12,18]^. Despite this 100-fold enrichment of EVs compared to HDL, the concentration of HDL after SEC2B will still be about four orders of magnitude higher than the estimated concentration of human plasma EVs^[4,19-21]^. Additionally, lipoproteins that overlap in diameter with EVs, such as chylomicrons and (V)LDL, are not or insufficiently separated from EVs by SEC2B^[20]^. In summary, after SEC, the isolated plasma EVs will also be incompletely separated from non-EV miRNA carriers such as lipoproteins and proteins, and their presence may interfere with the analyses of EV-associated miRNA.

Recently, multiple new SEC columns, resins, and combinations of resins were developed. As summarized in Table 1, these SEC columns vary in production method, resin pore size (about 70 nm for SEC2B, and 35 nm for SEC4B)^[22,23]^, degree of agarose cross-linking^[20,23]^, and column length^[22,23]^. In addition, a dual-mode chromatography (DMC) column has recently been introduced, which combines size-based and cation-exchange-based fractionations in one column^[20]^. The extent to which these different SEC columns affect the recovery and purity of EVs (i.e., the concentration of EVs per unit lipoprotein or protein) has never been extensively and systematically studied^[12,21,24]^. Additionally, it remains unclear how the presence of non-EV miRNA carriers affects the analysis of EV-associated miRNA.

**Table 1:** Overview of the characteristics, EV yield^*^, EV recovery^**^ and EV purity^***^ for each SEC column

		**Production method**	**Pore size (nm)**	**Cross-linking degree**	**Length (mL)**	**Resin type**	**EV-enriched fractions**	EV yield^*^ (EVs/mL)	EV recovery^**^ (%)	EV purity^***^ (ratio *vs.*)		
										**ng ApoA**	**ng ApoB**	**µg Protein**
**SEC2B**	Custom-made	Custom	70	CL	10	A	F8-10	1.13 × 10^7^	38.3	2.71 × 10^5^	4.86 × 10^4^	8.32 × 10^5^
	qEV Gen1	Commercial	70	CL	10	A	F7-9	9.45 × 10^6^	31.5	1.11 × 10^5^	1.28 × 10^4^	2.95 × 10^7^
	qEV Gen2 10 mL	Commercial	70	E-CL	10	A	F8-10	7.94 × 10^6^	26.6	2.34 × 10^5^	2.88 × 10^4^	7.94 × 10^7^
	qEV Gen2 14 mL	Commercial	70	E-CL	14	A	F10-12	7.76 × 10^6^	26.3	4.50 × 10^5^	5.59 × 10^4^	7.76 × 10^7^
	DMC2B	Custom	70	CL	12	A + FG	F10-12	3.51 × 10^6^	11.8	1.82 × 10^5^	4.98 × 10^4^	1.18 × 10^7^
**SEC4B**	Custom-made	Custom	35	CL	10	A	F8-10	1.61 × 10^7^	55.3	3.26 × 10^3^	2.90 × 10^2^	4.33 × 10^4^
	qEV Gen1	Commercial	35	CL	10	A	F7-9	1.44 × 10^7^	49.8	6.08 × 10^3^	4.74 × 10^2^	8.55 × 10^4^
	qEV Gen2 10 mL	Commercial	35	E-CL	10	A	F8-10	9.20 × 10^6^	31.9	4.16 × 10^4^	7.47 × 10^3^	2.61 × 10^7^
	qEV Gen2 14 mL	Commercial	35	E-CL	14	A	F10-12	9.09 × 10^6^	30.4	6.19 × 10^4^	1.23 × 10^4^	6.17 × 10^7^
	DMC4B	Custom	35	CL	12	A + FG	F9-11	4.43 × 10^6^	15.3	3.19 × 10^3^	3.08 × 10^3^	4.97 × 10^4^

^*^The term “EV yield” refers to the concentration of EVs in the pooled SEC fractions (diameter range 200-650 nm); ^**^the percentage “EV recovery” refers to the number of EVs in the pooled EV-enriched SEC fractions divided by the number of EVs in the starting material (platelet-depleted plasma) × 100%; ^***^the term “EV purity” refers to the concentration of EVs per unit protein or lipoprotein; A: agarose; CL: cross-linked; E-CL: extra cross-linked; EV: extracellular vesicle; F: fraction; FG: Fractogel; SEC: size-exclusion chromatography.

Therefore, this study investigates how SEC columns affect the recovery and purity of EVs isolated from human plasma, and determines its effects on the miRNA cargo analysis of EVs.

## METHODS

This section provides a summary of the methods used. Full details of the methods can be found in the supplementary information [Supplementary File 1].

### Blood collection and plasma preparation

Blood collection (EDTA, *n* = 5 healthy donors) and all further experimental protocols were approved by and performed in accordance with the guidelines of the Medical Ethical Committee of the Amsterdam Medical Centre, University of Amsterdam (W19_271#19.421). Informed consent was obtained from all participants. Plasma was prepared as described earlier^[7]^. Plasma was pooled and filtered to remove remaining platelets^[8,10]^. Aliquots of the pooled plasma samples were stored at -80 °C until further use. All experiments were performed using this pooled plasma sample, and therefore, all obtained data can be directly compared to each other.

### SEC

SEC and DMC columns were either commercially acquired (Izon Science, Christchurch, New Zealand) or custom-made (GE Healthcare, Uppsala, Sweden).

### Flow cytometry measurements of EVs

Concentrations of erythrocyte- [CD235a-Fluorescein isothiocyanate (FITC)], leukocyte- (CD45-Allophycocyanin), and platelet-derived (CD61-FITC) EV subpopulations [EV diameter range: 200-650 nm and Refractive Index (RI) < 1.42] were measured by flow cytometry using a calibrated Apogee A60-Micro flow cytometer (Apogee flow systems, Hemel Hempstead, UK). All flow cytometry experiments were reported in accordance with MIFlowCyt-EV^[25]^. Details can be found in the MIFlowCyt-EV document added to the supplementary information [Supplementary File 2].

### Lipoprotein and protein measurements

The ApoA-I and ApoB lipoprotein and protein concentrations were measured using an ELISA (R&D Systems, Abingdon, UK) and Bradford assay (Pierce^TM^ Coomassie Plus Assay Reagent, Thermo Fisher Scientific), respectively.

### miRNA isolation and qRT-PCR analysis

Total RNA was isolated using the miRNeasy serum/plasma kit (QIAgen, Hilden, Germany). RNA was reverse transcribed using the TaqMan® MicroRNA Reverse Transcription kit (Thermo Fisher Scientific) in a multiplex reaction containing primers for hsa-let7a-5p (Thermo Fisher Scientific, assay ID 000377), hsa-miR-21-5p (assay ID 000397), and hsa-miR-122-5p (assay ID 002245). Three µl of cDNA was subjected to 40 PCR cycles on an ABI 7500 Fast system. Considering that the sample input for RNA isolation was the same for all columns, the reported quantity of miRNAs is relative between the column types.

### Data analysis and statistics

A one-way analysis of variance (ANOVA) with Tukey’s multiple comparisons test was used to compare the mean of the (unmatched) groups. A least-squares linear regression analysis was used to study the relationship between the quantity of miRNAs and the log-transformed concentration of EVs, lipoproteins, and total protein. A *P*-value ≤ 0.05 was considered significant.

## RESULTS

### Determining the EV-enriched fractions

For each SEC column, the three fractions containing the highest concentration of EVs were identified using flow cytometry. Supplementary Figure 1A-B shows the measured concentration of EVs per fraction of each column. For each column, the three fractions containing the highest EV concentration were pooled before further analyses.

### Recovery and purity of EVs

**Figure 1:**
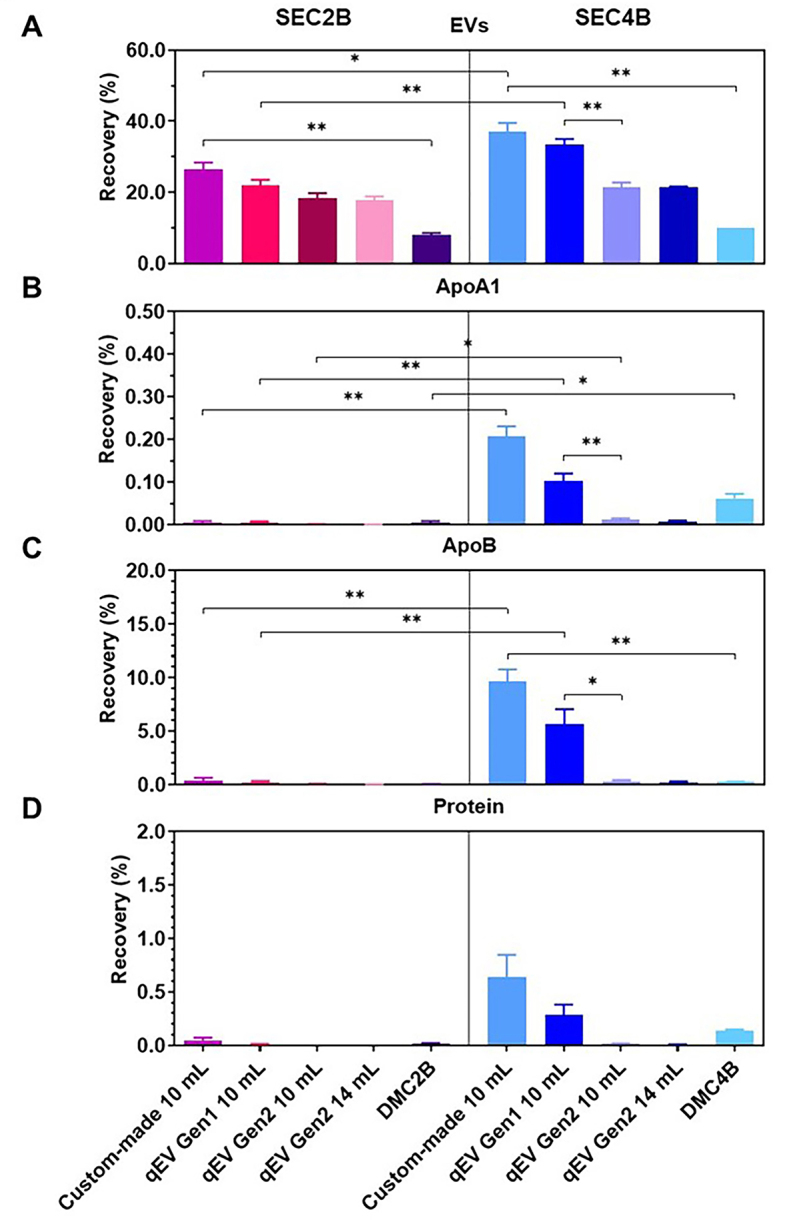
The recovery (%) of EVs^*^ (A), ApoA1 (B), ApoB (C), and Protein (D) in the pooled SEC fractions compared to the starting material (platelet-depleted plasma). EVs were measured by flow cytometry, ApoA1 (HDL) and ApoB [(V)LDL and chylomicrons] by ELISA, and protein by Bradford Assay. Experiments were performed in triplicate using platelet-depleted pooled plasma obtained from healthy controls. The starting material contained 4.3 × 10^7^ EVs, 2.4 × 10^6^ ng ApoA1, 5.9 × 10^5^ ng ApoB, and 6.8 × 10^4^ µg protein per mL of plasma. Adjusted p-values (one-way ANOVA with Tukey’s multiple comparisons test) obtained from assessing the statistical differences in EVs between SEC columns can be found in Supplementary Table 1. A *P*-value ≤ 0.05 was considered significant, and has been indicated with asterisks in the figure (^*^P < 0.05; ^**^P < 0.01). ^*^EVs were measured using flow cytometry (Apogee A60-Micro) and the concentration of EVs was calculated as the sum of particles that were positively labeled for CD61, CD235a, or CD45, with a size range of 200-650 nm and a refractive index < 1.42; DMC: dual-mode chromatography; EV: extracellular vesicle; SEC2B: size-exclusion chromatography 2B; SEC4B: size-exclusion chromatography 4B.

Figure 1A and Supplementary Table 1 show that the EV recovery, compared to the starting material - platelet-depleted human plasma, depends on resin pore size (70 nm *vs.* 35 nm), degree of agarose cross-linking (Gen1 *vs.* Gen2), and combination of resins (DMC *vs.* SEC). A 35 nm pore size yields a relatively high EV recovery of 33%-37%, especially for the Gen1 (i.e., legacy) columns, which have a lower degree of cross-linking compared to Gen2 columns. With 8%-10% recovery compared to the starting material, EV recovery was lowest for the DMC columns. The recovery of EVs was unaffected by the column production method and column length.

Figure 1B-D show the presence of ApoA1 (HDL), ApoB [(V)LDL and chylomicrons], and proteins in the pooled SEC fractions per column. All columns removed > 99.3% of ApoA1 (HDL) and proteins compared to the starting material. The efficacy of ApoB removal [(V)LDL and chylomicrons] mostly depended on the resin pore size. For example, 70 nm pore size columns removed > 99.6% of ApoB from the starting material compared to > 90.3% for the 35 nm pore size columns. The efficacy of lipoprotein and protein removal was unaffected by the column production method, degree of agarose cross-linking, column length, and resin combination. The absolute concentrations of EVs, ApoA, ApoB, and protein in the pooled SEC fractions per column are summarized in Supplementary Figure 2.

**Figure 2:**
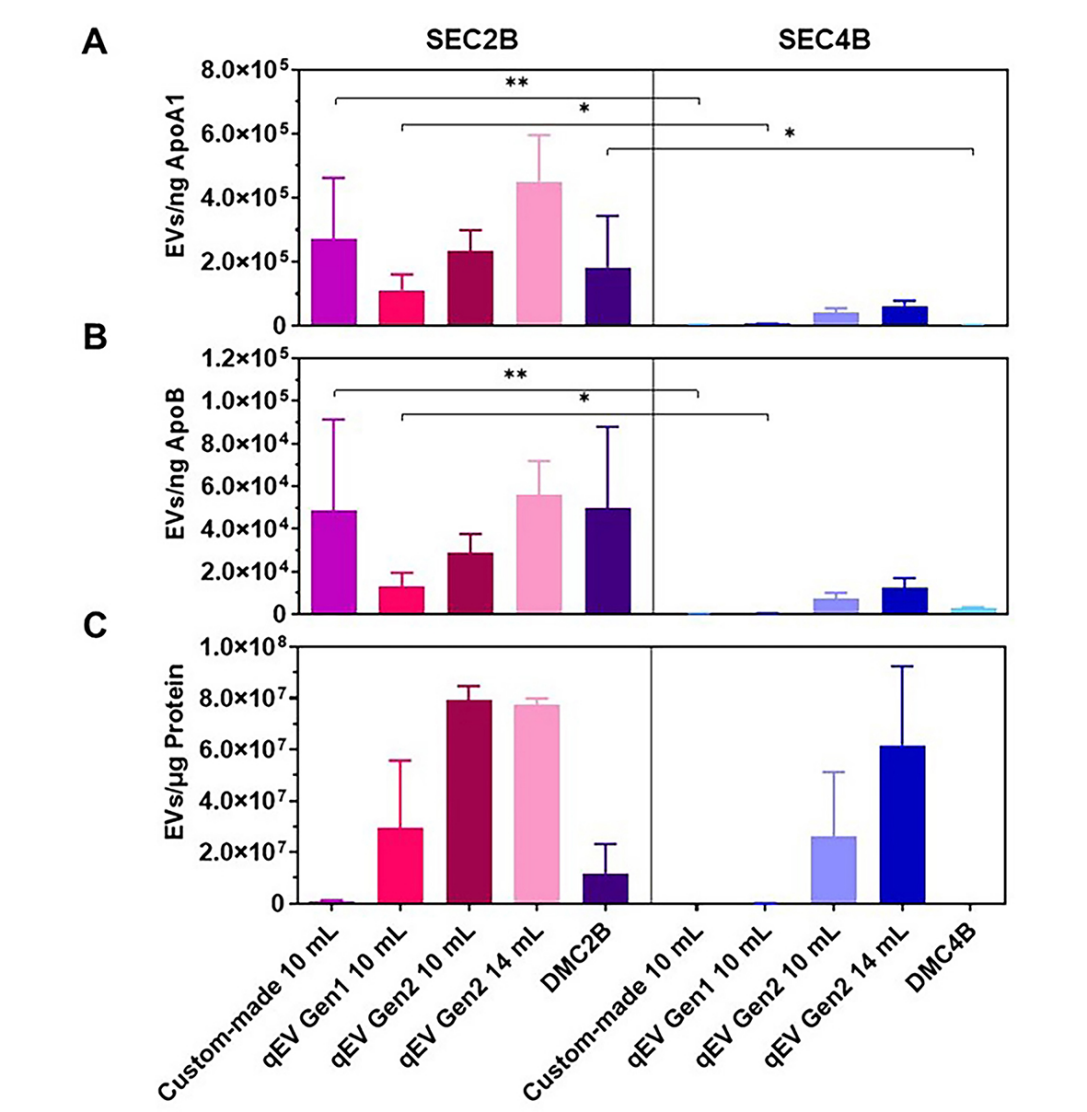
The purity of EVs^*^ in the pooled SEC fractions, compared to ApoA1 (HDL) by ELISA (A), ApoB [(V)LDL and chylomicrons] by ELISA (B), and protein as measured by Bradford Assay (C). Experiments were performed in triplicate using pooled plasma obtained from healthy controls. Adjusted *P*-values (one-way ANOVA with Tukey’s multiple comparisons test) obtained from assessing the statistical differences in EV purity between SEC columns can be found in Supplementary Table 1. A *P*-value ≤ 0.05 was considered significant, and has been indicated with asterisks in the figure (^*^P <0.05; ^**^P < 0.01). ^*^EVs were measured using flow cytometry (Apogee A60-Micro) and the concentration of EVs is calculated as the sum of particles that were positively labeled for CD61, CD235a, or CD45, with a size range of 200-650 nm and a refractive index < 1.42; DMC: dual-mode chromatography; EV: extracellular vesicle; SEC2B: size-exclusion chromatography 2B; SEC4B: size-exclusion chromatography 4B.

The relative EV purity, i.e., the concentration of EVs expressed per unit ApoA, ApoB, or protein, is shown in Figure 2A-C and Supplementary Table 1. The ratios of EVs/ApoA, EVs/ApoB, and EVs/protein increase 34-fold, 44-fold, and 120-fold when using 70 nm pore size columns compared to 35 nm columns, respectively. Thus, using 70 nm pore size resins increases the purity of EVs through more efficient removal of non-EV miRNA carriers. The EV purity was unaffected by the column production method, degree of agarose cross-linking, column length or by combining resins. Supplementary Figure 3 provides an overview of the ranking of all investigated columns in terms of EV yield and EV purity compared to lipoproteins.

### Effect of SEC column and resin variation on EV miRNA analysis

To understand how the presence of non-EV miRNA carriers affects the analysis of plasma EV-associated miRNA, we measured the relative quantity of three selected miRNAs in the pooled SEC fractions of all columns. The selected miRNAs are thought to be associated with EVs (let7a-5p)^[26]^, with EVs and protein (miR-21-5p)^[26]^, or with lipoproteins (miR-122-5p)^[27]^, and therefore can be considered as a read-out of the presence of EVs, protein, and lipoprotein.

Supplementary Figure 4A-C and Supplementary Table 2 show the qRT-PCR cycle thresholds (Ct) of each miRNA in the pooled SEC fractions of all columns. A lower Ct means a relatively higher miRNA quantity. The detected miRNA quantity is affected by the resin pore size and resin combination, with a two-fold higher miRNA quantity for both the 35 nm pore size columns compared to 70 nm pore size columns, and for the single resin columns compared to the DMC columns. In contrast, the column production method, agarose cross-linking, and column length do not affect the relative quantity of the three miRNAs. Thus, the columns with a small pore size and a single resin yield a higher quantity of miRNA. Besides a higher miRNA quantity, these columns have a higher EV yield but also a higher concentration of lipoproteins and proteins (i.e., lower EV purity). Therefore, it is still unknown if the increase in miRNA quantity is derived from EVs or from other non-EV miRNA carriers.

### Source of miRNA signals in plasma

**Figure 3:**
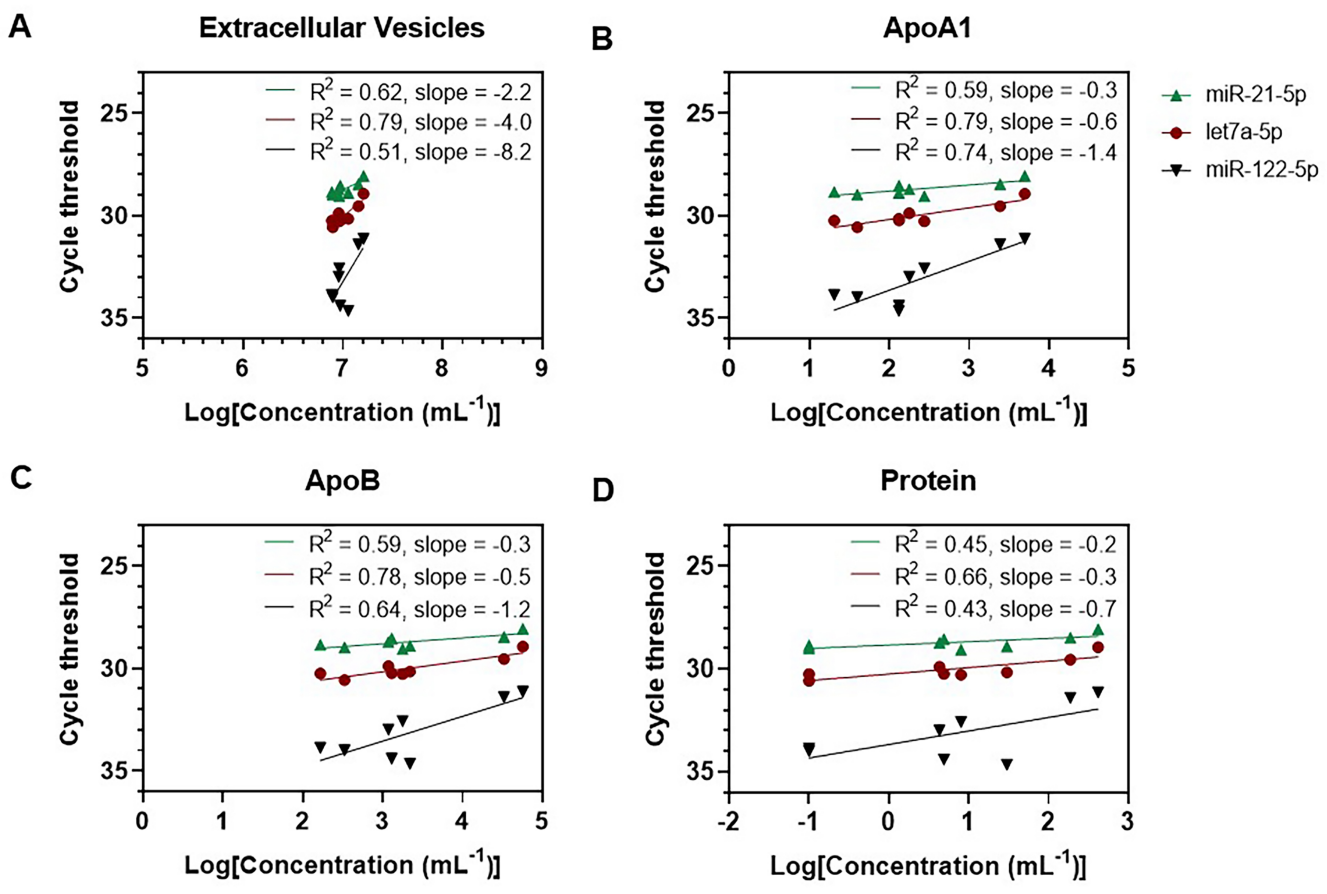
Regression analysis between the log-transformed concentration of EVs^*^ (A), ApoA1 (HDL) (B), ApoB ((V)LDL and chylomicrons) (C), and proteins (D) in the pooled SEC fractions, and the cycle threshold of two EV-associated (miR-21-5p and let7a-5p) and one lipoprotein-associated (miR-122-5p) miRNA, as detected by qRT-PCR. The slopes indicate the cycle threshold change per order of magnitude increase of the concentration. Experiments were performed in triplicate using pooled plasma obtained from healthy controls. A linear regression analysis was used to quantify the relationship between the relative quantity of miRNAs and the log-transformed concentration of EVs and (lipo)proteins. ^*^EVs were measured using flow cytometry (Apogee A60-Micro) and the concentration of EVs is calculated as the sum of particles that were positively labeled for CD61, CD235a, or CD45, with a size range of 200-650 nm and a refractive index < 1.42; EV: Extracellular vesicle; SEC: size-exclusion chromatography; qRT-PCR: quantitative real-time PCR.

To investigate whether the increase in miRNA quantity is derived from EVs or non-EV miRNA carriers, we used a least-squares linear regression analysis to study the relationship between the relative quantity of miRNAs and the concentration of EVs, ApoA, ApoB, and protein in the pooled SEC fractions for each column. The negative slopes of the fits in Figure 3A-D show that the quantity of miRNA correlates with the concentration of EVs (R^2^ = 0.51 to 0.79, slope = -2.2 to -8.2), ApoA1 (R^2^ = 0.59 to 0.79, slope = -0.3 to -1.4), ApoB (R^2^ = 0.59 to 0.78, slope = -0.3 to -1.2) and protein (R^2^ = 0.43 to 0.66, slope = -0.2 to -0.7). Thus, the quantity of miRNA increases with an increase in concentration of EVs, ApoA, ApoB, and protein. The steepest slope, and thus the strongest correlation, is present between the quantity of miRNAs and the concentration of EVs. In other words, an increase in the concentration of EVs increases the miRNA quantity more than an increase in the concentration of ApoA, ApoB, or proteins. The stronger correlation between the concentration of EVs and the quantity of the investigated miRNAs suggests that all three miRNAs are more strongly associated with EVs than with lipoproteins or proteins. The strength of the associations between the miRNAs and the EVs, lipoproteins, or proteins was also assessed using a Spearman correlation coefficient and can be found in Supplementary Figure 5.

### Effect of SEC column re-use on EV yield and purity

We tested the effect of re-using a qEV 70 nm Gen2 10 mL column up to five times on EV recovery and purity, with washing steps in between as recommended by the manufacturer. The details and results of the column re-use experiments are shown in the supplementary information [Supplementary File 3], Supplementary Figure 6A-E, and Supplementary Table 3. While re-using columns did not affect the EV and EV-miRNA recovery, a 4-to-5-fold increase in ApoA and ApoB concentration and a 2.5-fold increase in the protein concentration was observed after the second use in the pooled EV-enriched fractions. Thus, re-using 70 nm Gen2 10 mL SEC columns lowers the EV purity due to a less efficient separation from lipoproteins and proteins, but does not affect the quantity of the investigated miRNAs.

## DISCUSSION

In the present study, the resin pore size was the only column characteristic that affected all measured parameters, i.e., EV recovery, EV purity, and the quantity of (EV-associated) miRNA, as shown in Table 2. The EV recovery increased by about 8% when using 35 nm resin pore size columns compared to 70 nm columns. However, the 35 nm columns were also less efficient in the removal of lipoproteins and proteins compared to the 70 nm columns, which was most apparent with regard to the removal of ApoB (up to 9% difference). This higher ApoB concentration in the 35 nm columns resulted in a reduced EV purity, which is the number of EVs per unit lipoprotein or protein. Moreover, downstream analysis of miRNA showed that columns with a 35 nm pore size have a two-fold increase in the relative concentration of miRNAs compared to 70 nm columns.

**Table 2:** Overview of the effect of column characteristics on EV recovery^*^, lipoprotein and protein concentration, EV purity^**^ and on the detected quantity of miRNA

	**EV recovery**	**(Lipo)protein concentration**	**EV purity**	**Quantity of miRNA**
Production method Custom-made *vs.* commercially obtained	**-**	**-**	**-**	**-**
Pore size 35 nm *vs.* 70 nm	**↑**	**↑**	**↓**	**↑**
Cross-linking degree Low (Gen1) *vs.* high (Gen2)	**↑**	**-**	**-**	**-**
Column Length 10 mL *vs.* 14 mL	**-**	**-**	**-**	**-**
Resin combination DMC *vs.* SEC	**↓**	**-**	**-**	**↓**

Arrows indicate tendencies, and the results of statistical testing can be found in Supplementary Tables 1 and 2. ^*^“EV recovery” refers to the number of EVs (diameter range 200-650 nm) in the pooled EV-enriched SEC fractions divided by the number of EVs in the starting material (platelet-depleted plasma) × 100%; ^**^the term “EV purity” refers to the concentration of EVs per unit protein or lipoprotein. Effects are indicated with symbols, where “-” means no observed effect, an upward arrow means an increase, and a downward arrow means a decrease; EV: extracellular vesicle; DMC: dual-mode chromatography; SEC: size-exclusion chromatography.

A similar trend was observed for the columns where size- and cation-exchange-based fractionations were combined (i.e., DMC). Compared to DMC columns, SEC columns had an 18%-27% higher EV recovery and also showed a two-fold higher miRNA quantity. These increased miRNA quantities could be either due to the increased concentration of EVs or due to the inefficient removal of lipoproteins and proteins. Regression analysis shows that an increase in EV concentration affects the miRNA quantity more than an increase in the concentration of ApoA, ApoB, or protein, suggesting that the investigated miRNAs are more strongly associated with EVs than with lipoproteins or proteins. This was also confirmed when assessing the effect of consecutive column use, where an increase in lipoprotein and protein concentrations did not affect the quantity of the investigated miRNAs. This unexpected finding may be explained by the difficulty in particle separation in the current and previous studies. The investigated miRNAs are thought to be associated with EVs (let-7a-5p), EVs and proteins (miR-21-5p), or lipoproteins (miR-122-5p). However, as shown herein, pure isolation of EVs, lipoproteins, or proteins is difficult if not impossible. Therefore, mixtures of particles are typically analyzed, and associations between miRNAs and EVs, proteins, or lipoproteins should be interpreted with care. The columns with a low degree of cross-linking (Gen1) also show an increase in EV recovery compared to Gen2 columns, especially in the case of 35 nm pore size columns (12% increase). However, this increase does not significantly affect the detected quantity of miRNA. Nevertheless, it should be noted that the difference in EV recovery between Gen1 and Gen2 columns implies that switching column types during an ongoing study is not recommended. Finally, column production method and column length do not affect any of the measured parameters.

Four other studies compared the effects of resin pore size and column length on EV yield and purity^[18,21,24,28]^. Our results confirm that 35 nm columns increase the EV yield compared to 70 nm columns^[18,24,28]^. Similar to the results published by Ter-Ovanesyan *et al*.^[18,24,28]^, we show that columns with a higher EV yield have a reduced EV purity. In contrast, three studies reported that smaller resin pore sizes increased both the yield and the purity of EVs^[18,21,28]^. There are several possible explanations for these inconsistent findings. Firstly, in some of the studies, EV purity was only calculated relative to proteins and not to lipoproteins, while our results show that ApoB is more difficult to remove than proteins. Secondly, a limitation of this study is that with our protein quantification method, we measure the total concentration of protein, which includes both soluble proteins and proteins that are associated with EVs and lipoproteins, meaning that we may underestimate the amount of soluble protein that has been removed. There are other limitations to the current study. With the used flow cytometer settings and applied analysis, we measure EVs with a diameter range of 200-650 nm and a refractive index < 1.42^[29]^. Although this means that we do not measure all the EVs that are present in our samples, by excluding particles with a refractive index > 1.42, we do exclude false-positive lipoproteins that may bind antibodies and thus result in overestimation of the column performance. In addition, in this manuscript, we have not assessed the possible effect of different SEC columns on specific EV subtypes (e.g., platelet-derived *vs.* erythrocyte-derived EVs). This topic is currently being investigated in a follow-up study. Finally, it has been shown that proteins and lipoproteins can be present in the so-called corona on the surface of EVs^[30,31]^. Therefore, we cannot exclude the possibility that part of the (lipo)protein recovery in EV-enriched SEC fractions is derived from (lipo)proteins that are adsorbed to the surface of EVs.

Besides the choice of a SEC column, other possibilities to improve EV purity include (1) collecting blood samples from fasting donors^[4]^; and (2) combining isolation methods, such as size-based separation (e.g., SEC) followed by density-based separation (density gradient centrifugation). SEC separates EVs from the bulk of smaller lipoproteins such as HDL and soluble proteins, whereas density gradient centrifugation separates EVs from similar-sized lipoproteins, such as (V)LDL and chylomicrons^[4,16]^. However, combining several isolation methods will lower the EV yield.

In some studies, SEC columns are used multiple times. Although the manufacturer and Gaspar *et al*.^[16]^ mention that SEC columns can be used up to five times, with a cleaning step in between, we found little evidence in the literature to support these statements. Guo *et al*.^[18]^ only assessed the particle concentration in PBS in between column uses. Our results show that re-using a qEV 70 nm Gen2 10 mL column reduces the EV purity already after the second column use. Lipoproteins and proteins are possibly incompletely removed by the recommended washing procedure after the first column use, and then co-elute together with the second sample during the second use. Therefore, qEV 70 nm Gen2 10 mL column re-use with the advised washing protocol should be avoided if lipoproteins and proteins are present and thus may affect the downstream analyses. The increase in lipoprotein and protein concentrations did, however, not affect the quantity of detected miRNAs, confirming that the investigated miRNAs are indeed more associated with EVs than with lipoproteins and proteins. These results may vary depending on the washing steps performed, the type of starting material that is used (e.g., plasma, conditioned cell culture medium), the type of SEC column used, and the type of downstream analysis.

In conclusion, SEC-based EV isolation is a trade-off between EV recovery and EV purity, defined as the separation efficacy of non-EV miRNA carriers in the present study, and may affect downstream miRNA analysis. There are striking differences regarding the performance of the different SEC columns in EV recovery and in the separation of EVs from lipoproteins and proteins, which should be taken into account when choosing a SEC column type for a specific study. The three miRNAs investigated in this study are more strongly associated with EVs than with lipoproteins or proteins, suggesting that the presence of lipoproteins and proteins may be negligible for the studied miRNAs. However, the effect of these non-EV particles on the detection of other miRNAs is unknown. Thus, for specific EV-associated miRNA analysis, we recommend using either SEC columns with a 70 nm pore size due to their efficacy in removing non-EV miRNA carriers, or assessing the effect of non-EV particles on the quantity of miRNAs of interest.

## DECLARATIONS

### Availability of data and materials

The data supporting the main findings of this study are available at: https://doi.org/10.6084/m9.figshare.c.6368625.

### Copyright

© The Author(s) 2024.
