# Supplementary material for: Choice of size-exclusion chromatography column affects recovery, purity, and miRNA cargo analysis of extracellular vesicles from human plasma
Source: Extracell Vesicles Circ Nucl Acids. 2024 Sep 6;5(3):497–508. doi: 10.20517/evcna.2024.34 (PMC11648517; doi:10.20517/evcna.2024.34)
Supplement: Supplementary file 1 [file evcna-5-3-497-SupplementaryMaterials.pdf]

Supplementary Materials

Choice of size-exclusion chromatography column affects recovery, purity, and miRNA cargo analysis of extracellular vesicles from human plasma

Jillian W.P. Bracht[1,2,3], Mandy Los[1], Edwin van der Pol[1,2,3,4], Sandra A.W.M. Verkuijlen[2,5], Monique A.J. van Eijndhoven[2,5], D. Michiel Pegtel[2,5], Rienk Nieuwland[1,2,3]

[1]Department of Clinical Chemistry, Laboratory of Experimental Clinical Chemistry, Amsterdam Vesicle Center, Amsterdam UMC, location University of Amsterdam, Amsterdam 1105 AZ, The Netherlands.

[2]Cancer Centre Amsterdam, Imaging and Biomarkers, Amsterdam UMC, location University of Amsterdam, Amsterdam 1105 AZ, The Netherlands.

[3]Amsterdam Cardiovascular Sciences, Atherosclerosis and Ischemic Syndromes, Amsterdam UMC, location University of Amsterdam, Amsterdam 1105 AZ, the Netherlands.

[4]Department of Biomedical Engineering and Physics, Amsterdam UMC, location University of Amsterdam, Amsterdam 1105 AZ, The Netherlands.

[5]Department of Pathology, Amsterdam UMC, location Vrije Universiteit Amsterdam, Amsterdam 1081 HV, The Netherlands.

Correspondence to: Dr. Jillian W.P. Bracht, Department of Clinical Chemistry, Laboratory of Experimental Clinical Chemistry, Amsterdam Vesicle Center, Amsterdam University Medical Center, location University of Amsterdam, Meibergdreef 9, Room: B1-238, Amsterdam 1105 AZ, The Netherlands. E-mail: j.w.p.bracht@amsterdamumc.nl

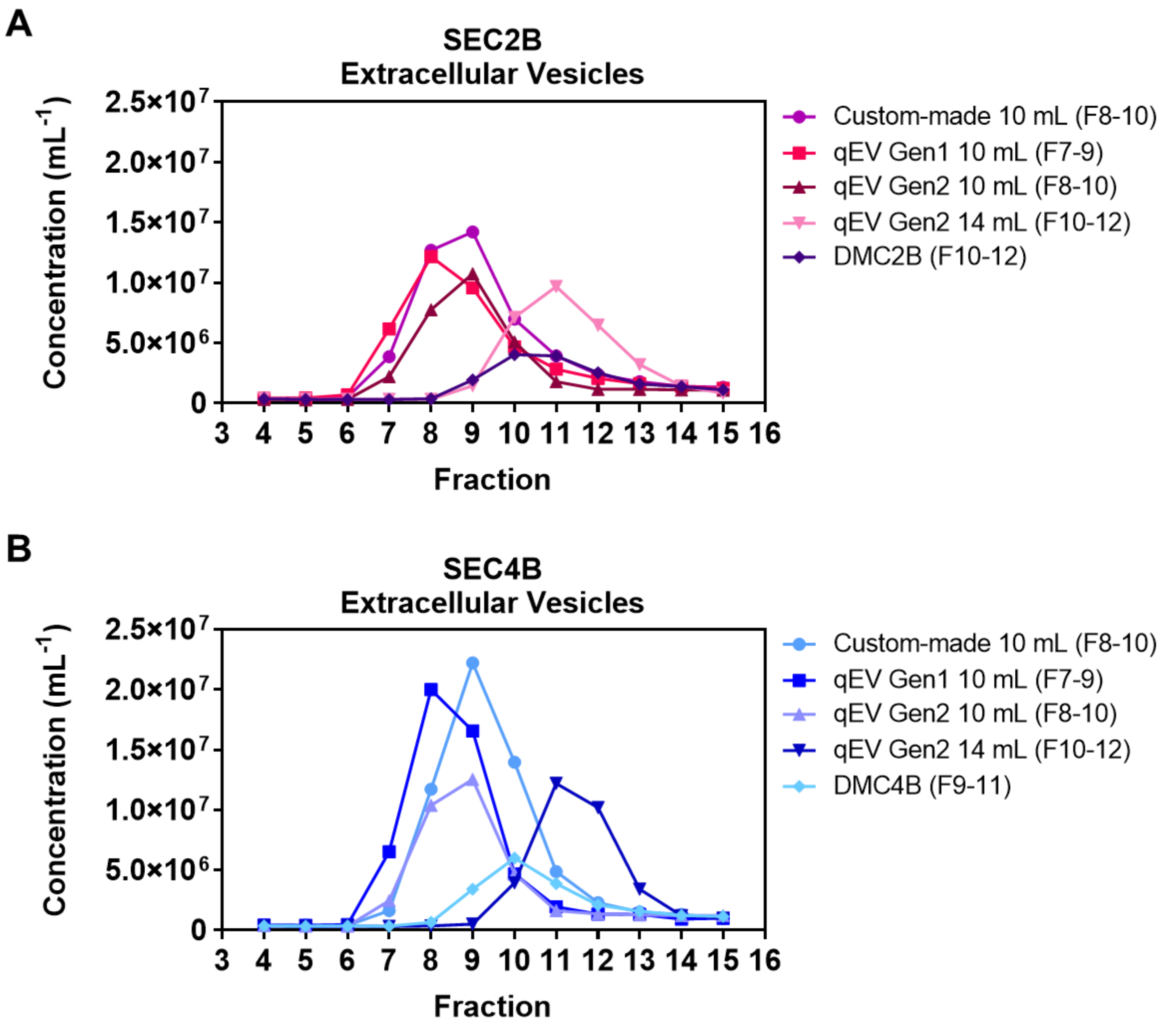

Supplementary Figure 1. The concentration of EVs* in fractions 4-15 (each 0.5 mL) for SEC2B or 70 nm pore size columns (A) and SEC4B or 35 nm pore size columns (B). For each column, the three fractions that contained the highest EV concentrations, as indicated between brackets, were pooled and used for further analyses. Experiments were performed in triplicate using pooled plasma obtained from healthy controls. *EVs were measured using flow cytometry (Apogee A60-Micro) and the concentration of EVs is calculated as the sum of particles that were positively labeled for CD61, CD235a or CD45, with a size range of 200-650 nm and a refractive index < 1.42; EV: Extracellular vesicle; DMC: dual-mode chromatography; SEC2B: size-exclusion chromatography 2B; SEC4B: size-exclusion chromatography 4B.

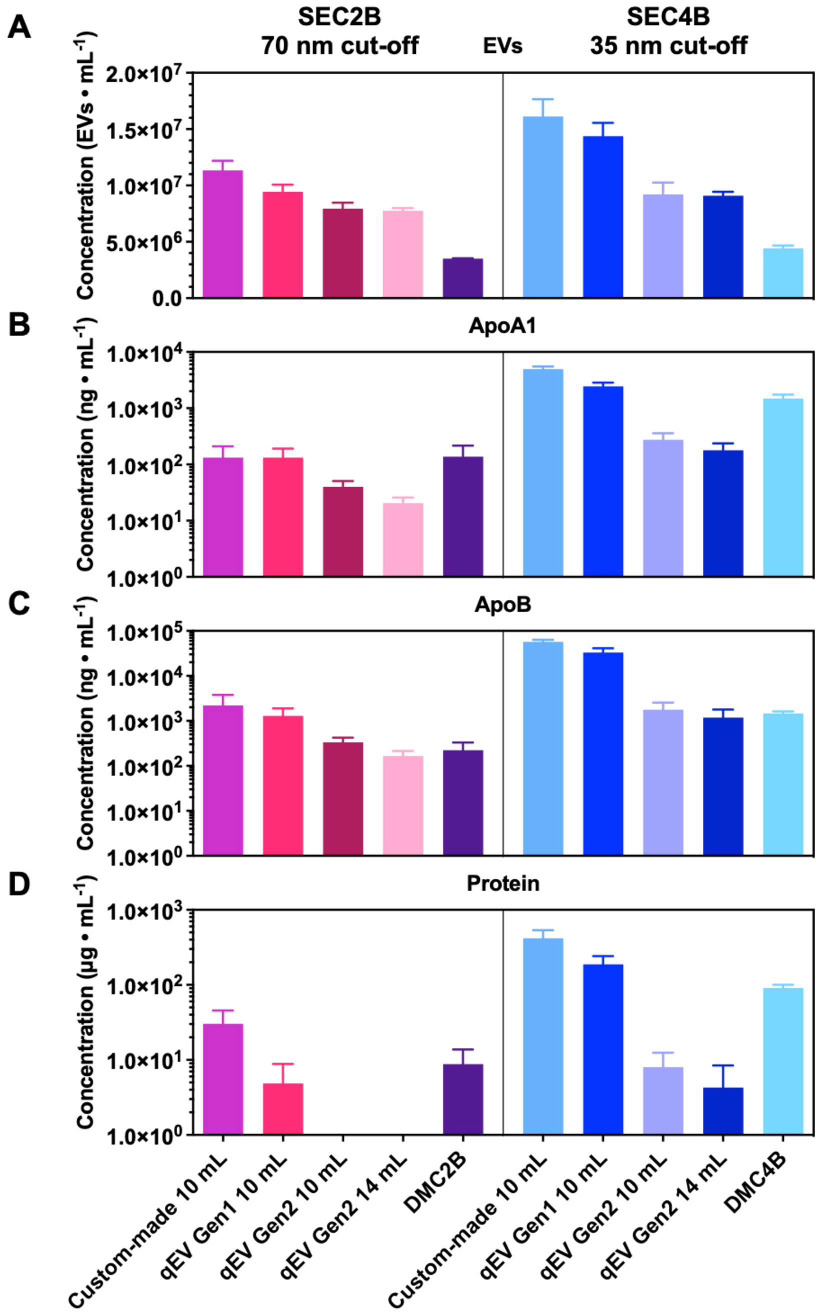

Supplementary Figure 2. The concentration (A) of EVs* (linear axis), (B-C) lipoproteins (logarithmic axes), and (D) proteins (logarithmic axis) in the pooled SEC fractions. EVs were measured by flow cytometry, ApoA1 (HDL) and ApoB [(V)LDL and chylomicrons] by ELISA, and protein by Bradford Assay. Experiments were performed in triplicate using pooled plasma obtained from healthy controls. *EVs were measured using flow cytometry (Apogee A60-Micro) and the concentration of EVs is calculated as the sum of particles that were positively labeled for CD61, CD235a or CD45, with a size range of 200-650 nm and a refractive index < 1.42.; DMC: Dual-mode chromatography; EV: extracellular vesicle; SEC2B: size-exclusion chromatography 2B; SEC4B: size-exclusion chromatography 4B.

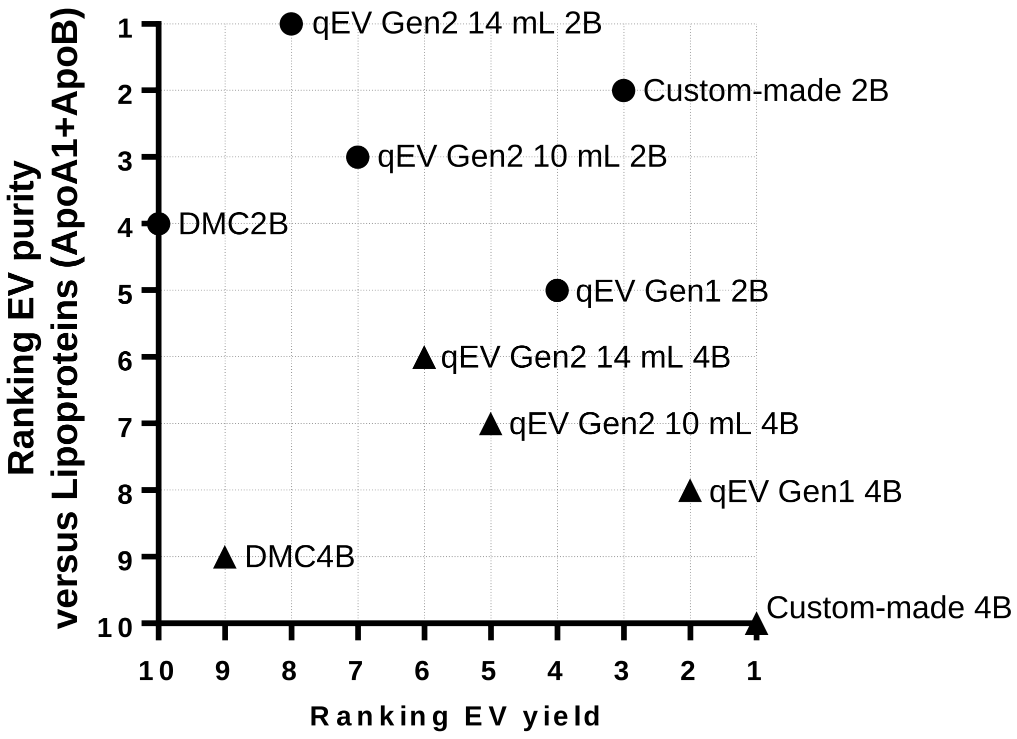

Supplementary Figure 3. Ranking of all investigated SEC columns based on EV yield (x-axis), and EV purity (ratio EVs versus lipoproteins ApoA1 and ApoB, y-axis) in the pooled SEC fractions. The columns with the highest (1) and lowest (10) EV yield and EV purity were ranked. The EV and lipoprotein concentrations per column can be found in Supplementary Figure 2, and the ratios of EVs versus lipoproteins per column can be found in Figure 2. The data points depicted as circles indicate the columns with a 70 nm pore size (SEC2B), the data points depicted as triangles indicate the columns with a 35 nm pore size (SEC4B). *EVs were measured using flow cytometry (Apogee A60-Micro) and the concentration of EVs is calculated as the sum of particles that were positively labeled for CD61, CD235a or CD45, with a size range of 200-650 nm and a refractive index < 1.42; DMC: Dual-mode chromatography; EV: extracellular vesicle; SEC2B: size-exclusion chromatography 2B; SEC4B: size-exclusion chromatography 4B.

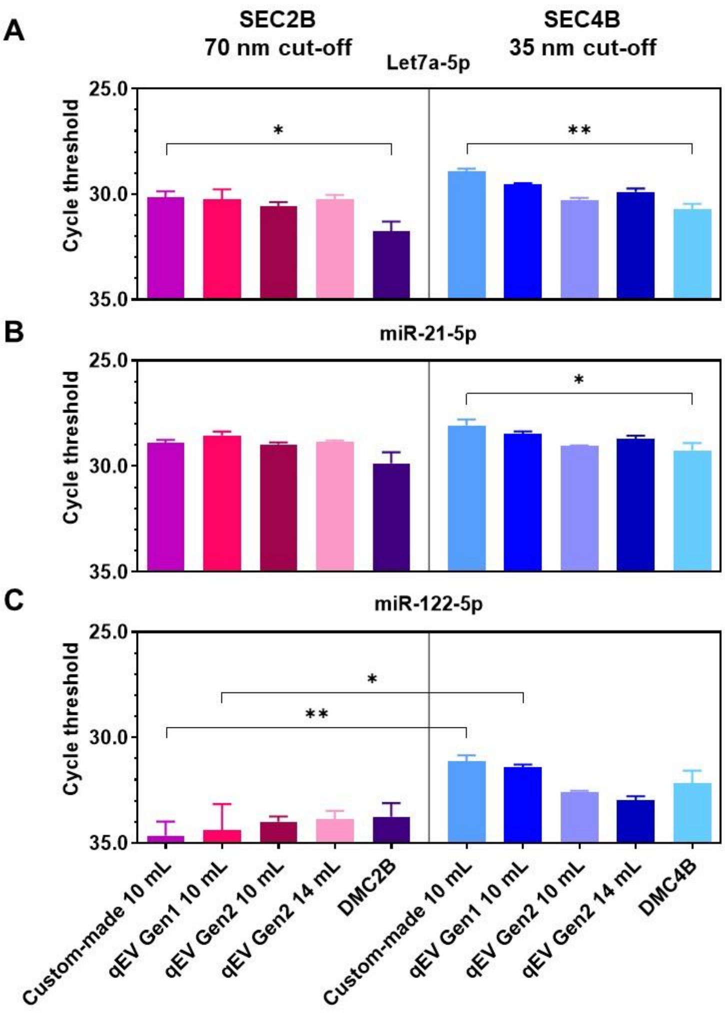

Supplementary Figure 4. Cycle thresholds measured by qRT-PCR for the detection of EV-associated miRNA let7a-5p (A) and miR-21-5p (B), and lipoprotein-associated miR-122-5p (C) in the pooled SEC fractions of all investigated columns. Experiments were performed in triplicate using pooled plasma obtained from healthy controls. Adjusted *P*-values (one-way ANOVA with Tukey's multiple comparisons test) obtained from assessing the statistical differences in miRNA quantities between SEC columns can be found in Supplementary Table 2. A *P*-value ≤ 0.05 was considered significant, and has been indicated with asterisks in the figure (*: $P < 0.05$; **: $P < 0.01$). [*]EVs were measured using flow cytometry (Apogee A60-Micro) and the concentration of EVs is calculated as the sum of particles that were positively labeled for CD61, CD235a or CD45, with a size range of 200-650 nm and a refractive index < 1.4; DMC: Dual-mode chromatography; EV: extracellular vesicle; SEC2B: size-exclusion chromatography 2B; SEC4B: size-exclusion chromatography 4B; qRT-PCR: quantitative real-time PCR.

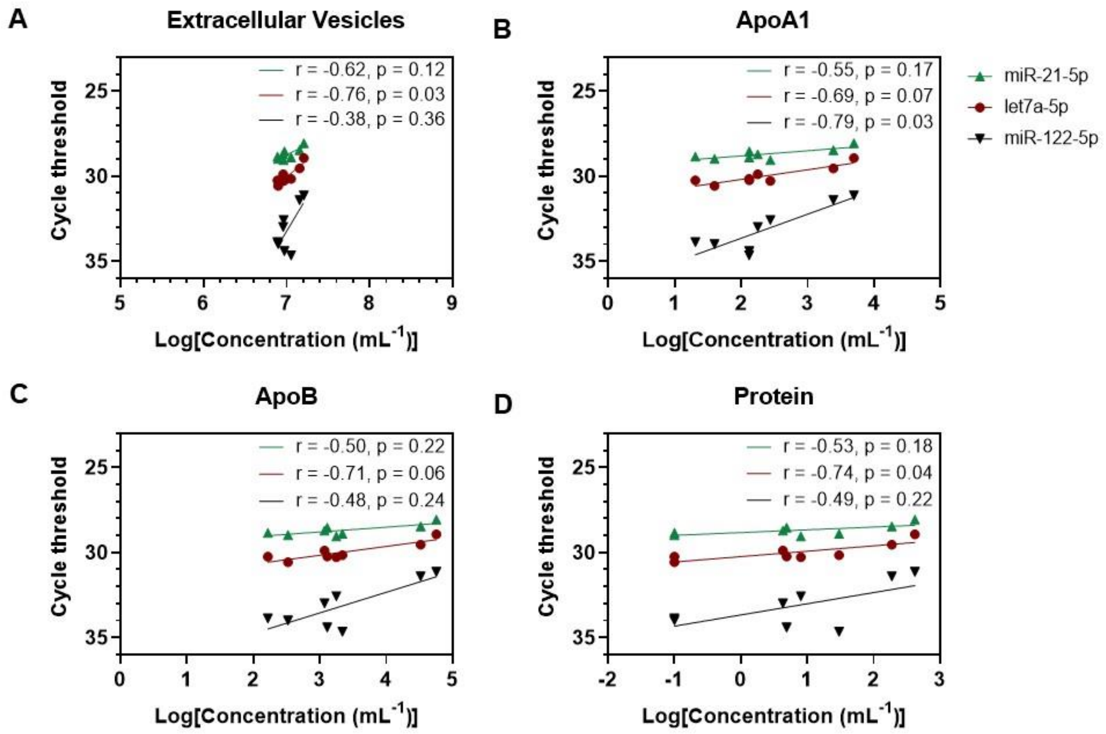

Supplementary Figure 5. Spearman correlation analysis between the log-transformed concentration of EVs* (A), ApoA1 (HDL) (B), ApoB [(V)LDL and chylomicrons] (C), and proteins (D) in the pooled SEC fractions, and the cycle threshold of two EV-associated (miR-21-5p and let7a-5p) and one lipoprotein-associated (miR-122-5p) miRNA, as detected by qRT-PCR. The strength of the relationship between the relative miRNA quantities and particle concentration is indicated by the spearman correlation coefficient (r) and the corresponding *P*-value. Experiments were performed in triplicate using pooled plasma obtained from healthy controls. *EVs were measured using flow cytometry (Apogee A60-Micro) and the concentration of EVs is calculated as the sum of particles that were positively labeled for CD61, CD235a or CD45, with a size range of 200-650 nm and a refractive index < 1.42; EV: Extracellular vesicle; SEC: size-exclusion chromatography; qRT-PCR: quantitative real-time PCR.

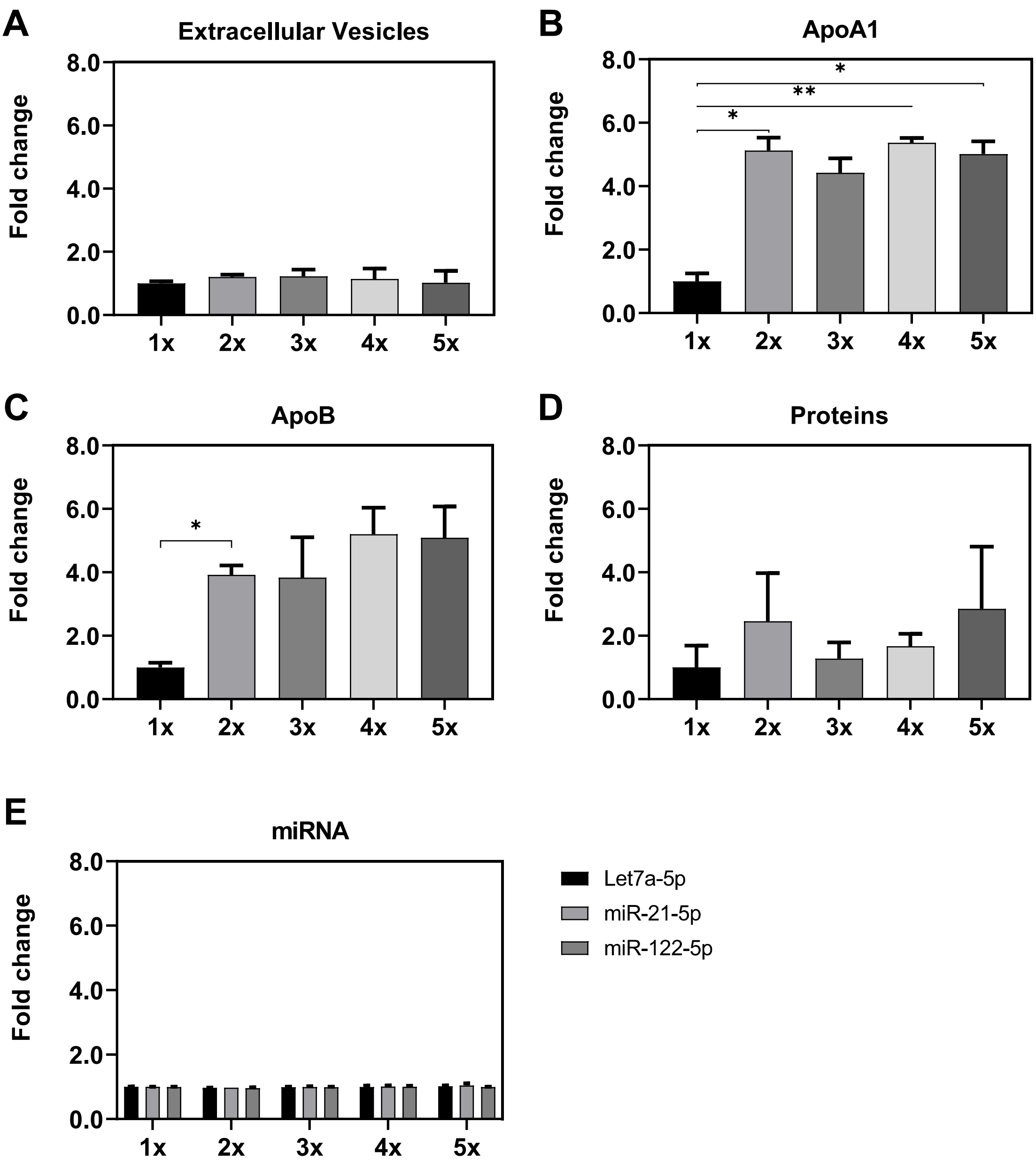

Supplementary Figure 6. The effect of re-using a qEV 70 nm Gen2 10 mL column up to five times, using aliquots of the same plasma sample, on EV(-RNA) yield and purity in the pooled SEC fractions. Columns were washed in between uses, according to the manufacturer's instructions. Fold changes in EV* concentration (A), lipoprotein concentration (B-C) protein concentration (D) and RNA quantity (E) after consecutive column use compared to the first-time use were calculated. Experiments were performed in triplicate using pooled plasma obtained from healthy controls. A repeated measures ANOVA (with a Geisser-Greenhouse correction) was carried out to compare the mean of the matched groups, and results can be found in Supplementary Table 3. A *P*-value ≤ 0.05 was considered

significant, and has been indicated with asterisks in the figure (*: $P < 0.05$; **: $P < 0.01$). *EVs were measured using flow cytometry (Apogee A60-Micro) and the concentration of EVs is calculated as the sum of particles that were positively labeled for CD61, CD235a or CD45, with a size range of 200-650 nm and a refractive index < 1.42; EV: Extracellular vesicle.

Supplementary Table 1. Fold changes (FC) and adjusted p-values (one-way ANOVA with Tukey's multiple comparisons test) obtained from assessing the statistical differences in EV* recovery (data shown in Figure 1) and the ratio of EVs per unit lipoprotein or protein between the assessed SEC columns (data shown in Figure 2)

		**EVs**		**EVs/ng ApoA1**		**EVs/ng ApoB**		**EVs/µg Protein**	
**Production method**	Custom-made 10 mL 2B *vs.* qEV Gen1 10 mL 2B	FC = 1.2	*P* = 0.57	FC = 2.4	*P* > 0.99	FC = 3.8	*P* > 0.99	FC = 0.03	*P* = 0.70
	Custom-made 10 mL 4B *vs.* qEV Gen1 10 mL 4B	FC = 1.1	*P* = 0.97	FC = 0.5	*P* > 0.99	FC = 0.6	*P* > 0.99	FC = 0.5	*P* > 0.99
**Pore size**	Custom-made 10 mL 2B *vs.* Custom-made 10 mL 4B	FC = 0.7	*P* = 0.02	FC = 83.0	*P* < 0.01	FC = 167.6	*P* < 0.01	FC = 19.2	*P* = 0.72
	qEV Gen1 10 mL 2B *vs.* qEV Gen1 10 mL 4B	FC = 0.7	*P* < 0.01	FC = 18.3	*P* = 0.03	FC = 27.1	*P* = 0.02	FC = 344.8	*P* = 0.10
	qEV Gen2 10 mL 2B *vs.* qEV Gen2 10 mL 4B	FC = 0.9	*P* = 0.76	FC = 5.6	*P* = 0.33	FC = 3.9	*P* = 0.70	FC = 3.0	*P* = 0.52

	qEV Gen2 14 mL 2B vs qEV Gen2 14 mL 4B	FC = 0.9	*P* = 0.47	FC = 7.3	*P* = 0.20	FC = 4.6	*P* = 0.54	FC = 1.3	*P* = 0.99
	DMC2B *vs.* DMC4B	FC = 0.8	*P* = 0.25	FC = 57.1	*P* = 0.02	FC = 16.2	*P* = 0.23	FC = 236.6	*P* = 0.38
**Degree of cross-linking**	qEV Gen1 10 mL 2B *vs.* qEV Gen2 10 mL 2B	FC = 1.2	*P* = 0.53	FC = 0.5	*P* = 0.96	FC = 0.5	*P* = 0.95	FC = 0.4	*P* = 0.78
	qEV Gen1 10 mL 4B *vs.* qEV Gen2 10 mL 4B	FC = 1.6	*P* < 0.01	FC = 0.2	*P* = 0.29	FC = 0.1	*P* = 0.07	FC = 0.01	*P* = 0.23
**Length**	qEV Gen2 10 mL 2B *vs.* qEV Gen2 14 mL 2B	FC = 1.0	*P* > 0.99	FC = 0.5	*P* > 0.99	FC = 0.5	*P* > 0.99	FC = 1.0	*P* > 0.99
	qEV Gen2 10 mL 4B *vs.* qEV Gen2 14 mL 4B	FC = 1.0	*P* > 0.99	FC = 0.7	*P* > 0.99	FC = 0.6	*P* > 0.99	FC = 0.4	*P* = 0.98
**Resin combination**	Custom-made 10 mL 2B *vs.* DMC2B	FC = 3.2	*P* < 0.01	FC = 1.5	*P* = 0.92	FC = 1.0	*P* > 0.99	FC = 0.1	*P* > 0.99
	Custom-made 10 mL 4B *vs.* DMC4B	FC = 3.6	*P* < 0.01	FC = 1.0	*P* > 0.99	FC = 0.1	*P* = 0.13	FC = 0.9	*P* > 0.99

A *P*-value ≤ 0.05 was considered significant. *EVs were measured using flow cytometry (Apogee A60-Micro) and the concentration of EVs is calculated as the sum of particles that were positively labeled for CD61, CD235a or CD45, with a size range of 200-650 nm and a

refractive index < 1.42; EV: Extracellular vesicle; FC: fold change; SEC: size-exclusion chromatography.

Supplementary Table 2. Difference in cycle threshold (Ct) and adjusted *P*-values (one-way ANOVA with Tukey's multiple comparisons test) obtained from assessing the statistical differences in miRNA quantities between SEC columns (data shown in Supplementary Figure 4).

		**Let7a-5p**		**miR-21-5p**		**miR-122-5p**	
**Production method**	Custom-made 10 mL 2B *vs.* qEV Gen1 10 mL 2B	ΔCt = -0.07	$P > 0.99$	ΔCt = 0.36	$P = 0.98$	ΔCt = 0.25	$P > 0.99$
	Custom-made 10 mL 4B *vs.* qEV Gen1 10 mL 4B	ΔCt = -0.62	$P = 0.78$	ΔCt = -0.39	$P = 0.96$	ΔCt = -0.28	$P > 0.99$
**Pore size**	Custom-made 10 mL 2B *vs.* Custom-made 10 mL 4B	ΔCt = 1.24	$P = 0.07$	ΔCt = 0.82	$P = 0.33$	ΔCt = 3.53	$P < 0.01$
	qEV Gen1 10 mL 2B vs qEV Gen1 10 mL 4B	ΔCt = 0.69	$P = 0.70$	ΔCt = 0.07	$P > 0.99$	ΔCt = 3.00	$P = 0.02$
	qEV Gen2 10 mL 2B *vs.* qEV Gen2 10 mL 4B	ΔCt = 0.30	$P > 0.99$	ΔCt = -0.07	$P > 0.99$	ΔCt = 1.42	$P = 0.70$
	qEV Gen2 14 mL 2B *vs.* qEV Gen2 14 mL 4B	ΔCt = 0.35	$P = 0.99$	ΔCt = 0.12	$P > 0.99$	ΔCt = 0.90	$P = 0.97$
	DMC2B *vs.* DMC4B	ΔCt = 1.05	$P = 0.23$	ΔCt = 0.62	$P = 0.73$	ΔCt = 1.59	$P = 0.56$
**Degree of cross-linking**	qEV Gen1 10 mL 2B *vs.* qEV Gen2 10 mL 2B	ΔCt = -0.34	$P = 0.99$	ΔCt = -0.44	$P = 0.94$	ΔCt = 0.41	$P > 0.99$
	qEV Gen1 10 mL 4B *vs.* qEV Gen2 10 mL 4B	ΔCt = -0.72	$P = 0.64$	ΔCt = -0.58	$P = 0.77$	ΔCt = -1.17	$P = 0.84$

**Length**	qEV Gen2 10 mL 2B *vs.* qEV Gen2 14 mL 2B	ΔCt = 0.33	*P* > 0.99	ΔCt = 0.14	*P* > 0.99	ΔCt = 0.11	*P* > 0.99
	qEV Gen2 10 mL 4B *vs.* qEV Gen2 14 mL 4B	ΔCt = 0.38	*P* = 0.99	ΔCt = 0.33	*P* = 0.99	ΔCt = -0.41	*P* > 0.99
**Resin combination**	Custom-made 10 mL 2B *vs.* DMC2B	ΔCt = -1.60	*P* = 0.01	ΔCt = -0.97	*P* = 0.20	ΔCt = 0.91	*P* = 0.97
	Custom-made 10 mL 4B *vs.* DMC4B	ΔCt = -1.78	*P* < 0.01	ΔCt = -1.17	*P* = 0.05	ΔCt = -1.03	*P* = 0.92

A *P*-value ≤ 0.05 was considered significant. ΔCt: Difference in cycle threshold; SEC: size-exclusion chromatography.

Supplementary Table 3. Fold changes (FC) and adjusted *P*-values (repeated measures ANOVA with a Geisser-Greenhouse correction, and Dunnett's multiple comparisons test) obtained from assessing the statistical differences in EV (-RNA), lipoprotein and protein concentrations after consecutive column use compared to the first time use (data shown in Supplementary Figure 5)

	**EVs**		**ApoA1**		**ApoB**		**Proteins**		**Let7a-5p**		**miR-21-5p**		**miR-122-5p**	
**2x *vs.* 1x**	FC = 1.2	*P* = 0.17	FC = 5.1	*P* = 0.01	FC = 3.9	*P* = 0.04	FC = 2.5	*P* = 0.45	FC = 0.97	*P* = 0.50	FC = 0.98	*P* = 0.11	FC = 0.96	*P* = 0.45
**3x *vs.* 1x**	FC = 1.2	*P* = 0.56	FC = 4.4	*P* = 0.09	FC = 3.8	*P* = 0.36	FC = 1.3	*P* = 0.54	FC = 0.99	*P* = 0.42	FC = 0.99	*P* > 0.99	FC = 0.99	*P* = 0.84
**4x *vs.* 1x**	FC = 1.1	*P* = 0.93	FC = 5.4	*P* < 0.01	FC = 5.2	*P* = 0.10	FC = 1.7	*P* = 0.36	FC = 1.01	*P* = 0.92	FC = 1.01	*P* = 0.98	FC = 1.00	*P* > 0.99
**5x *vs.* 1x**	FC = 1.0	*P* > 0.99	FC = 5.0	*P* = 0.02	FC = 5.1	*P* = 0.13	FC = 2.9	*P* = 0.54	FC = 1.02	*P* = 0.69	FC = 1.04	*P* = 0.84	FC = 0.99	*P* = 0.90

A P-value ≤ 0.05 was considered significant. *EVs were measured using flow cytometry (Apogee A60-Micro) and the concentration of EVs is calculated as the sum of particles that were positively labeled for CD61, CD235a or CD45, with a size range of 200-650 nm and a RI < 1.42; EV: Extracellular vesicle; FC: fold change; RI: refractive index.

Supplementary File 1. Materials and Methods

BLOOD COLLECTION AND PLASMA PREPARATION

Blood collection was in accordance with the guidelines of the Medical Ethical Committee of the Amsterdam Medical Centre, University of Amsterdam (W19_271#19.421). Blood was collected from five non-fasting healthy individuals. All donors denied having a disease and/or to use drugs and/or medication. Venous blood was collected using a 21-Gauge needle, and the first 2 mL of blood was discarded. Four tubes of Ethylene Diamine Tetra Acetic acid (EDTA) blood (6 mL; BD Biosciences, San Jose, CA) were collected from each donor and processed within 15 minutes after collection[25].

Plasma was prepared according to the protocol published by the International Society on Thrombosis and Haemostasis (ISTH)[7], using a Rotina 380 R centrifuge (Hettich, Tuttlingen, Germany). Whole blood was centrifuged for 15 minutes at 2,500 x g at 20 °C, with the centrifuge acceleration set at 9, and deceleration set at 1. The obtained plasma was collected to exactly 10 mm (using a lego brick) above the buffy coat, and transferred to a 15 mL polypropylene tube (Greiner Bio-One B.V., Alphen aan den Rijn, The Netherlands). The plasma was then centrifuged for 15 minutes at 2,500 x g and 20 °C, with the centrifuge acceleration set at 9, and deceleration set at 1. The obtained plasma was collected to exactly 10 mm above the cell pellet and plasma from all donors was pooled. To remove platelets and ery-ghosts from the plasma samples, polycarbonate, track-etched membrane filters with a pore diameter of 0.8 μm and a filter diameter of 25 mm (Isopore™, Merck Millipore, Darmstadt, Germany) were used[8,10]. Aliquots of the pooled plasma samples were stored at -80 °C until further use. All experiments were performed using this pooled plasma sample, and therefore all obtained data can be directly compared to each other.

SIZE-EXCLUSION CHROMATOGRAPHY COLUMNS

Size-exclusion chromatography (SEC) columns were commercially acquired (qEV original 70 nm, qEV original 35 nm, qEV Gen2 10 mL 70 nm, qEV Gen2 10 mL 35 nm, qEV Gen2 14 mL 70 nm and qEV Gen2 14 mL 35 nm; Izon Science, Christchurch, New Zealand) or

custom-made [Custom-made SEC2B, custom-made SEC4B, dual-mode chromatography (DMC) 2B and DMC4B].

For the custom-made SEC columns, Sepharose CL-2B (GE Healthcare, Uppsala, Sweden) or Sepharose CL-4B (Cytiva, Shrewsbury, MA) was washed with Dulbecco's Phosphate-Buffered Saline (DPBS; Thermo Fisher Scientific, Waltham, MA). Subsequently, the double 20 µm polyethylene frit on the bottom of a 15 mL Kinesis TELOS Chromatography Filtration Column (Cole-Parmer, St Neots, UK) was degassed, and the column was stacked with 10 mL washed Sepharose. Then, a separate 20 µm Kinesis TELOS polyethylene frit was degassed, and the frit was carefully placed on top of the stacked Sepharose. Columns were washed with DPBS containing 0.05% sodium azide and stored at 4 °C. Prior to use, columns were washed with DPBS to remove sodium azide.

The DMC columns were prepared as the custom-made SEC columns, but 2 mL Fractogel EMD SO3- (M) (Merck Millipore) was washed with DPBS and stacked into the column before adding Sepharose, as described by van Deun *et al.*[20]. DMC columns were stored at 4 °C without addition of sodium azide, and used within two days after preparation.

SIZE-EXCLUSION CHROMATOGRAPHY

For each SEC column, one mL of pooled plasma was thawed at 37 °C for 3 minutes. Plasma was loaded onto the SEC columns, and 0.5 mL fractions (F) were collected directly after sample loading. DPBS was used as running buffer. F1-3 (1.5 mL in total) were considered void fractions, and therefore discarded. F4-15 were collected and the concentration of EVs in each fraction was determined using calibrated flow cytometry. For each column, the three fractions containing the highest concentration of EVs (EV enriched fractions, Supplementary Figure 1 and Table 1) were pooled before further analyses.

EXTRACELLULAR VESICLE YIELD AND PURITY

Throughout the manuscript, the term "EV yield" refers to the concentration of EVs in the pooled EV-enriched SEC fractions (diameter range 200-650 nm). The term "EV purity" refers to the concentration of EVs per unit lipoprotein or protein. Finally, "EV recovery" [Table 1] refers to the percentage of EVs in the pooled SEC fractions compared to concentration of EVs in the starting material (platelet-depleted plasma).

FLOW CYTOMETRY MEASUREMENTS OF EXTRACELLULAR VESICLES

For the column comparison experiments, the concentration of erythrocyte- [anti-human CD235a-fluorescein isothiocyanate (CD235a-FITC), > 219 molecules of equivalent soluble fluorochrome (MESF)], leukocyte- [anti-human CD45-allophycocyanin (CD45-APC)], > 31 MESF) and platelet-derived (anti-human CD61-FITC, > 196 MESF) EV subpopulations [EV diameter range: 200-650 nm and Refractive Index (RI) < 1.42] were measured by flow cytometry using a calibrated A60-Micro flow cytometer (Apogee flow systems, Hemel Hempstead, UK). All flow cytometry experiments were reported in accordance with MIFlowCyt-EV[26]. Details can be found in the MIFlowCyt-EV document added to the Supplementary Information [Supplementary File 2].

PROTEIN MEASUREMENTS

The total protein concentration (detection range 100 – 1,500 µg/mL) of the pooled EV-enriched fractions was determined using a Bradford protein assay, according to manufacturer's instructions (Pierce™ Coomassie Plus Assay Reagent, Thermo Fisher Scientific). Absorbance was measured at 595 nm on a SpectraMax i3X plate reader (Molecular Devices, San Jose, CA).

LIPOPROTEIN MEASUREMENTS

The lipoprotein concentration (ApoA-I and ApoB) of the pooled EV-enriched fractions was determined by ELISA, according to manufacturer's instructions (R&D Systems, Abingdon, UK). Detection ranges for the ApoA-I and ApoB ELISA kits were 6.3-200 ng/mL and 39.1-2,500 ng/mL, respectively. Optical density was determined using a SpectraMax i3X plate reader (Molecular Devices) at 450 nm.

MIRNA ISOLATION AND QRT-PCR ANALYSIS

Total RNA was isolated from 200 µL EV-enriched SEC fractions using the miRNeasy serum/plasma kit (QIAgen, Hilden, Germany) according to the manufacturers' protocol. RNA was eluted in 14 µl nuclease free water, and reverse transcribed using the TaqMan® MicroRNA Reverse Transcription kit (Thermo Fisher Scientific) in a multiplex reaction containing RT-primers for hsa-let7a-5p (Thermo Fisher Scientific, assay ID 000377), hsa-miR-21-5p (Thermo Fisher Scientific, assay ID 000397) and hsa-miR-122-5p (Thermo Fisher Scientific, assay ID 002245). After cDNA synthesis, nuclease free water was added up to a final volume of 50 µl. Three µl of cDNA was subjected to 40 cycles of 95 °C for 15 seconds

and 60 °C for 1 minute on an ABI 7500 Fast system. All samples were measured in duplicate and data was analyzed using 7500 Software v2.0.6. Considering that the sample input for RNA isolation was the same for all columns, the reported quantity of miRNAs is relative between the column types.

DATA ANALYSIS AND STATISTICS

Flow cytometry data was processed using FlowJo (v10.8.1; FlowJo, Ashland, OR) and MATLAB (R2020b; Mathworks, Natick, MA). Lipoprotein and protein data was analyzed according to manufacturer's instructions, using Microsoft Excel (v2016; Microsoft Office, Redmond, WA). Statistical analyses were performed using Prism 9.0 (GraphPad, La Jolla, CA). A one-way ANOVA with Tukey's multiple comparisons test was used to compare the mean of the (unmatched) groups. A least square linear regression analysis was used to study the relationship between the quantity of miRNAs and the log-transformed concentration of EVs, lipoproteins and total protein in the pooled EV-enriched SEC fractions per column. A P-value ≤ 0.05 was considered significant.

Supplementary File 2. MIFlowCyt-EV checklist

FLOW CYTOMETRY

Experimental design

The purpose of this flow cytometry (A60-Micro, Apogee Flow Systems, Hemel Hempstead, UK) experiment was to quantify the concentration of extracellular vesicles (EVs) derived from platelets (CD61+), erythrocytes (CD235a+) and leukocytes (CD45+) in platelet-depleted plasma. EVs were isolated using size-exclusion chromatography (SEC) columns with different types of resins and column lengths. The goal of this experiment was to investigate the efficacy of multiple recently developed SEC columns and resins for their ability to separate EVs from lipoproteins and proteins, and study the downstream effects on miRNA analysis. EV yield was determined using calibrated flow cytometry. EV purity was defined as the amount of EVs per unit lipoprotein or protein in the EV-enriched fractions, which was measured using a Bradford protein assay (total protein concentration), ApoA1 (e.g., HDL) and ApoB [e.g., (V)LDL and chylomicrons] ELISAs. In addition, the effect of re-using SEC columns on EV yield and purity was tested according to the manufacturer's protocol.

To determine EV concentrations, all samples were measured with an Apogee A60-micro in a 96-well plate (one and a half plate per experiment), using an autosampler. Experiments were performed in triplicate, and each experiment contained antibody in buffer controls, corresponding to the labels included in this experiment, and a buffer-only control. Scatter calibration and flow rate calibration were performed on the day of the experiments. Fluorescence calibration was performed two to four months after the ‘column comparison’ and four months before the “re-using SEC columns” experiments. To automatically process data, determine optimal samples dilutions, apply calibrations, determine and apply gates, generate reports with scatter plots and generate data summaries, MATLAB R2020b (Mathworks, Natick, MA) was used.

Sample dilutions

As the particle concentration in plasma and EV samples differs between individuals and differs between the type of SEC column and resin used, samples require different dilutions to avoid swarm detection[1,2] while maintaining statistically significant counts within a reasonable measurement time. Although serial dilutions are recommended to find the optimal dilution, we consider serial dilutions unfeasible due to the large number of samples. Therefore, we developed a procedure to estimate the optimal sample dilution[2]. In sum, we showed that for our flow cytometer and settings used, a count rate $\leq 5.0 \cdot 10^3$ events/second unlikely results in swarm detection[3].

To find the dilution resulting in a count rate $\leq 5.0 \cdot 10^3$ events per second, we measured the total concentration of particles for 30 seconds without staining and calculated the minimum dilution required before staining (next section). Samples with a count rate $> 5.0 \cdot 10^3$ were diluted in Dulbecco’s Phosphate-buffered saline (DPBS, Corning, Corning, NY) and re-measured. The staining procedure adds an extra dilution of 11.3-fold to the overall dilution.

EV staining

Column comparison

For the column comparison experiments, EVs in plasma and EV-enriched SEC fractions were stained using anti-CD61-FITC (Dako Amstelveen, The Netherlands), anti-CD45-APC (Biolegend, San Diego, CA, USA), and anti-CD235a-FITC (Dako, Amstelveen, The Netherlands). Prior to staining, antibodies were pre-diluted in DPBS, as described in Table S1.1, and centrifuged at 18,890 x g for 5 minutes to remove aggregates. Two-and-a-half µl of

each antibody was incubated with 20 µl pre-staining diluted sample for 2 hours at room temperature in the dark. CD61 and CD45 were used for double staining, by adding a total of 5 µl antibody to each sample. Post-staining, samples were diluted to decrease background fluorescence of unbound fluorophores by adding 200 µl DPBS to each sample.

Re-using SEC columns

To study the effect of re-using SEC columns, a cocktail of anti-CD61-APC (Invitrogen, Waltham, MA), anti-CD45-APC (Biolegend) and anti-CD235a-APC (R&D systems, Abingdon, UK) was used to stain EVs in plasma and EV-enriched SEC fractions. Antibodies were pre-diluted in DPBS and centrifuged at 18,890 x g for 5 minutes, leaving 10 µl with antibody aggregates behind. Equal volumes of each antibody were mixed and 7.5 µl of this cocktail was incubated with 20 µl pre-staining diluted sample for 2 hours at room temperature in the dark. Post-staining, samples were diluted to decrease background fluorescence of unbound fluorophores by adding 200 µl DPBS to each sample.

Buffer-only control

Each experiment day, one DPBS sample was measured with the same flow cytometer and acquisition settings as all other samples.

Column comparison

The mean count rate was 81 events per second, which is lower than the target count rate (2.5-$5.0 \cdot 10^3$ events per second) for plasma and EV samples. The minimum count rate of buffer-only control was 48 events per second, the maximum count rate measured was 109 events per second. One of the five buffer-only controls was excluded due to a failed measurement.

Re-using SEC columns

Background events in buffer were estimated at an average of 45 events per second. With a maximum count rate of 96 events per second and a minimum count rate of 19 events per sec, background events are substantially lower than the count rate of plasma and EV samples (2.5-$5.0 \cdot 10^3$ events per second).

Buffer with reagents control

Each experiment day a buffer with reagent control was included for each reagent [Table S1.1], which was measured with the same flow cytometer and acquisition settings as all samples.

Column comparison

For anti-CD235a-FITC in buffer, an average of 46 events per second were measured. This is lower than in the buffer-only control (109 events per second). However, for anti-CD61-FITC combined with anti-CD45-APC in buffer the count rate was higher compared to buffer-only control, with 178 events per second. Of note, for both the anti-CD61-FITC combined with anti-CD45-APC and for the anti-CD235a-FITC in buffer control, one out of five control samples was excluded due to failed measurements. To investigate whether background counts caused by anti-CD61-FITC/anti-CD45-APC and anti-CD235a-FITC affect the reported results, we applied the same calibrations and gates to anti-CD61-FITC/anti-CD45-APC and anti-CD235a-FITC in buffer as to the plasma and EV samples stained with the corresponding antibody. On average, we obtained 159 CD61-FITC+ and 136 CD45-APC+ events in buffer, while the mean of CD61-FITC+ and CD45-APC+ events in plasma and EV fractions were 712 and 1729, respectively. For CD235a-FITC, we obtained an average of 181 CD235a-FITC+ events in buffer and an average of 967 CD235a+ events in plasma and EV samples. It is important to mention that the average of positive events in plasma and EV-enriched SEC fractions also includes samples without the bulk of EVs, which lowers the average. In conclusion, the numbers of positive background events are acceptable.

Re-using SEC columns

Antibody cocktail (anti-CD61/anti-CD45/anti-CD235a) in buffer showed a mean count rate of 100 events per second. This count rate is slightly higher than the buffer-only control (45 events per second). Not only a low count rate, but also 31 false APC positive events were detected, applying the same gates and calibrations to these antibody cocktail in buffer samples as to our stained plasma and EV samples. This amount of false positive events is minimal compared to the number of APC+ events in our samples, in which a minimum of 602 and a maximum of 5,091 APC+ events is detected (average of 2,842 APC+ events). The number of false APC positive events influences our results negligibly.

Unstained controls

Column comparison

Unstained plasma controls were measured with the same dilution and settings as the stained plasma samples. Unstained plasma samples had a minimum count rate of 2,048 events per

second and a maximum count rate of 3,308 events per second. These count rates are comparable with count rates during measurements.

Re-using SEC columns

Both unstained plasma and EV fractions were measured with the same dilution and settings as stained samples. Unstained plasma and EV fractions had average count rates of 3,288 and 1,713 events per second, respectively. For unstained plasma this average is comparable with count rates of stained plasma samples (3,241 evets/sec). The average of 1,713 events per second for unstained EV fractions was slightly lower compared to the average of stained EV fractions (2,456 events per second), however, acceptable.

Isotype controls

No isotype controls were included in this experiment, since the used antibodies did not show any binding to Fc-receptors in previous experiments[4].

Trigger channel and threshold

Based on the buffer-only control (109 events per second), the acquisition software was set up to trigger at 24 arbitrary units SSC, which is equivalent to a side scattering cross section of 6 nm^2 (Rosetta Calibration v1.28, Exometry, Amsterdam, The Netherlands).

Flow rate quantification

On the measurement days, we used 110-nm FITC beads with a specified concentration (Apogee calibration beads, Apogee Flow Systems, Hemel Hempstead, UK) to validate the flow rate of the A60-Micro flow cytometer. As the A60-Micro is equipped with a syringe pump with volumetric control, we assumed a flow rate of 3.01 μL/min for all measurements.

Fluorescence calibration

Calibration of the fluorescence detectors from arbitrary units (a.u.) to molecules of equivalent soluble fluorochrome (MESF) was accomplished using 2 µm APC Quantitation beads (2364-87, BD), and 2 µm FITC Quatitation Beads (2364-85, BD). Calibrations of the APC and FITC detectors were performed on 2021-03-01. ApoCAL was used to correct for variations in the fluorescent detectors. For each measurement, we added fluorescent intensities in MESF to the flow cytometry data files (MATLAB R2018a) using the following equation:

$\text{I(MESF)} = 10^{a \cdot \log_{10} \text{I(a.u.)} + b}$	Equation S1

where I, is the fluorescence intensity, and a and b are the slope and the intercept of the linear fits respectively, see Table S1.2.

Light scatter calibration

We used Rosetta Calibration (v1.29, Exometry BV, Amsterdam, The Netherlands) to relate scatter measured by forward scattering (FSC) and side scattering (SSC) to the effective scattering cross section and diameter of EVs. Figure S1.1 shows print screens of the scatter calibrations. We modelled EVs as core-shell particles with a core refractive index of 1.38, a shell refractive index of 1.48, and a shell thickness of 6 nm. For each measurement, we added the FSC and SSC cross sections and EV diameters to the cytometry data files. The SSC trigger threshold corresponds to a side scattering cross section of 6 nm^2.

EV refractive index approximation

Flow-SR was applied to determine the size and refractive index of particles and improve specificity by enabling label-free differentiation between EVs and lipoprotein particles[5,6]. Flow-SR was performed as previously described[5,6]. Lookup tables were calculated for diameters ranging from 10 to 1000 nm, with step sizes of 1 nm, and refractive indices from 1.35 to 1.80 with step sizes of 0.001. The diameter and refractive index of each particle was added to the .fcs file by custom-build software (MATLAB R2020b).

Because Flow-SR requires accurate measurements of both FSC and SSC, we applied Flow-SR only to particles with diameters > 200 nm and fulfilling the condition:

$\text{SSC}(\text{nm}^2) > -0.7 \cdot \text{FSC}(\text{nm}^2) + 3$	Equation S2

EV number concentration

The concentrations reported in the manuscript describe the number of particles (1) that exceeded the SSC threshold, corresponding to a side scattering cross section of 6 nm^2, (2) that were collected during time intervals, for which the count rate was within 750 counts/second above and below the mean count rate, (3) with a diameter > 200 nm as determined by Flow-SR[5], (4) fulfilling the condition of equation S2, (5) with a refractive index < 1.42 to omit false positively labelled lipoproteins, and (6) that were positive for APC, or FITC, per mL of plasma.

Data sharing

Data is available via: https://doi.org/10.6084/m9.figshare.c.6368625

REFERENCES

1. van der Pol E, van Gemert MJ, Sturk A, Nieuwland R, van Leeuwen TG. Single vs. swarm detection of microparticles and exosomes by flow cytometry. *J Thromb Haemost.* 2012;10(5):919-930.

2. Buntsma NC, Gasecka A, Roos Y, van Leeuwen TG, van der Pol E, Nieuwland R. EDTA stabilizes the concentration of platelet-derived extracellular vesicles during blood collection and handling. *Platelets.* 2022;33(5):764-771.

3. Buntsma NC, Shahsavari M, Gasecka A, Nieuwland R, van Leeuwen TG, van der Pol E. Preventing swarm detection in extracellular vesicle flow cytometry: a clinically applicable procedure. *Res Pract Thromb Haemost.* 2023;7(4):100171.

4. Gasecka A, Nieuwland R, Budnik M, et al. Ticagrelor attenuates the increase of extracellular vesicle concentrations in plasma after acute myocardial infarction compared to clopidogrel. *J Thromb Haemost.* 2020;18(3):609-623.

5. van der Pol E, de Rond L, Coumans FAW, et al. Absolute sizing and label-free identification of extracellular vesicles by flow cytometry. *Nanomedicine.* 2018;14(3):801-810.

6. de Rond L, Libregts S, Rikkert LG, et al. Refractive index to evaluate staining specificity of extracellular vesicles by flow cytometry. *J Extracell Vesicles.* 2019;8(1):1643671.

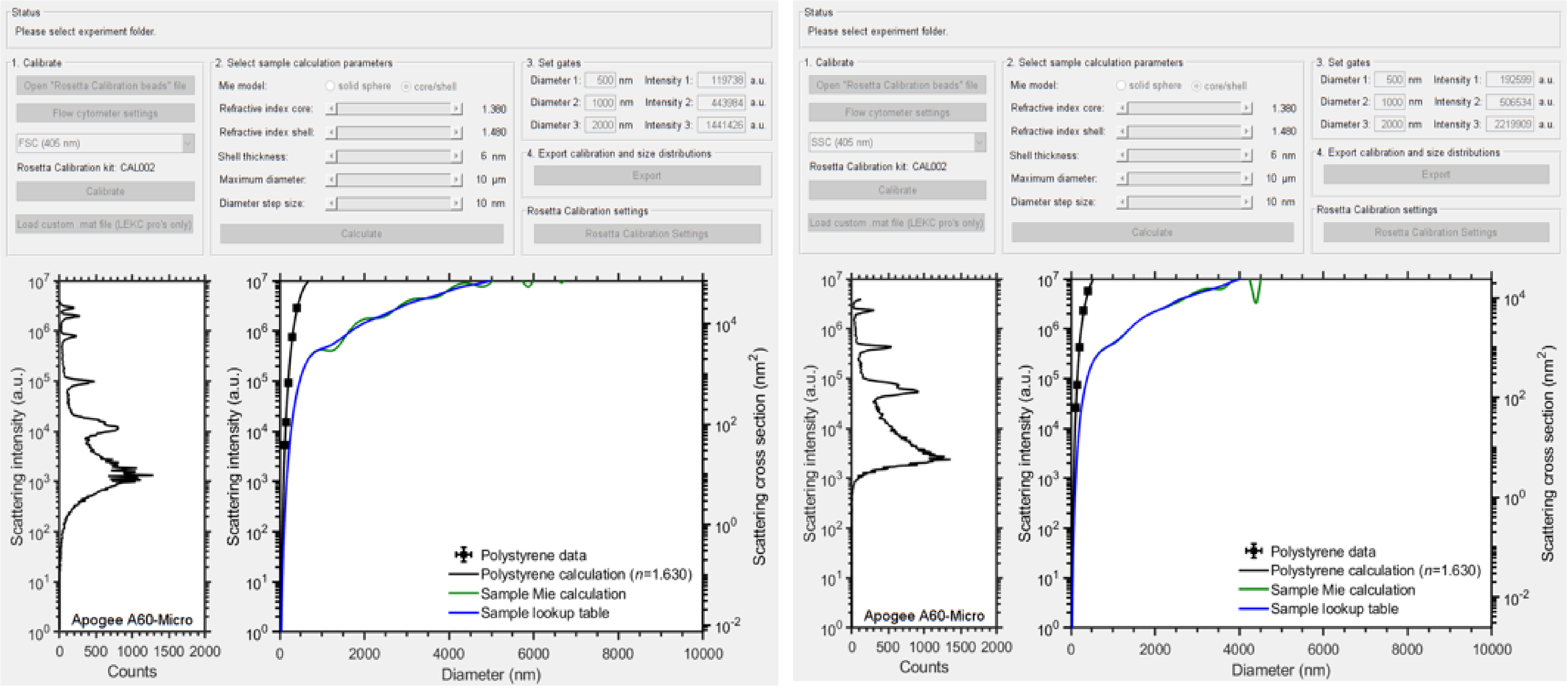

Figure S1.1. Rosetta Calibration to relate scatter to the diameter of EVs. Forward scatter and side scatter calibration of the A60-Micro by Rosetta Calibration. To relate scatter to the diameter of EVs, we modelled EVs as core-shell particles with a core refractive index of 1.38, a shell refractive index of 1.48, and a shell thickness of 6 nm.

Table S1.1. Overview of staining reagents. Characteristics being measured, analyte, analyte detector, reporter, isotype, clone, concentration, manufacturer, catalog number and lot number of used staining reagents. The antibody concentration during measurements was 11.3-fold lower than the antibody concentration during staining

Characteristic measured	Analyte	Analyte detector	Reporter	Isotype	Clone	Concentration during staining (µg mL^{-1})	Manufacturer	Catalog number	Lot number
<u>Column comparison</u>									
Integrin	Human CD61	Anti-human CD61 antibody	FITC	IgG1	Y2/51	1.25	Dako	F0803	20027302
Glycoprotein	Human CD235a	Anti-human CD235a antibody	FITC	IgG1	JC159	5.56	Dako	F0870	20064863
Glycoprotein	Human CD45	Anti-human CD45 antibody	APC	IgG1	HI30	0.23	Biolegend	304037	B272158
<u>Re-using SEC columns</u>									
Integrin	Human CD61	Anti-human CD61 antibody	APC	IgG1	VI-PL2	0.75	Invitrogen	17-0619-42	2311256
Glycoprotein	Human CD235a	Anti-human CD235a	APC	IgG1	R10	4.55	R&D systems	FAB12281A	ADGT022009

		antibody							
Glycoprotein	Human CD45	Anti-human CD45	APC	IgG1	HI30	0.20	Biolegend	304037	B311564

APC: Allophycocyanin; FITC: fluorescein isothiocyanate.

Table S1.2. Overview of fluorescence calibrations

	Calibration date	Slope	Intercept	R^2
Column comparison				
APC (2021-11-24)	2022-03-01	1.1936	-2.6546	0.9949
APC (2021-11-25)	2022-03-01	1.1936	-2.7289	0.9949
APC (2021-12-01)	2022-03-01	1.1936	-2.6997	0.9949
APC (2021-12-02)	2022-03-01	1.1936	-2.6560	0.9949
APC (2022-01-05)	2022-03-01	1.1936	-2.5089	0.9949
APC (2022-01-12)	2022-03-01	1.1936	-2.5089	0.9949
FITC	2022-03-01	1,1823	-2.1922	0.9993
Re-using SEC columns				
APC (2022-07-06)	2022-03-01	1.1936	-2.4812	0.9949
APC (2022-07-25)	2022-03-01	1.1936	-2.4870	0.9949
APC (2022-07-27)	2022-03-01	1.1936	-2.4972	0.9949

APC: Allophycocyanin; FITC: fluorescein isothiocyanate.

Supplementary File 3. Column Re-use

MATERIALS AND METHODS

A qEV Gen2 10 mL 70 nm SEC column (Izon Science) was used for five consecutive times. In between each use the column was washed with 8.5 mL sodium hydroxide (NaOH) and 17 mL dPBS, as recommended by the manufacturer. Five 1 mL aliquots of pooled platelet-free plasma were thawed at 37 °C for 3 minutes. For each use, 1 mL of plasma was loaded onto the column, and 0.5 mL fractions (F) were collected after sample loading. DPBS was used as running buffer. The three EV-enriched fractions (F8-10) were pooled for further analyses.

The concentration of EVs in the pooled SEC fractions after each column use was measured using flow cytometry. The concentration of erythrocyte- [anti-human CD235a-allophycocyanin (CD235a-APC)], leukocyte- (anti-human CD45-APC) and platelet-derived (anti-human CD61-APC) EVs [diameter range: 200-650 nm, > 72 molecules of equivalent soluble fluorochrome (MESF)] were measured by flow cytometry, using a calibrated A60-Micro flow cytometer (Apogee flow systems, Hemel Hempstead, UK). All flow cytometry

experiments were reported in accordance with MIFlowCyt-EV[1], and details can be found in the MIFlowCyt-EV document added to the supplements. Lipoprotein concentration (ApoA and ApoB) of the EV-enriched fractions after each column use were determined by ELISA, as described in the Materials and Methods section of the main manuscript. Total protein concentration was determined using a Bradford protein assay, and RNA was isolated from the pooled SEC fractions as described in the Materials and Methods section of the main manuscript.

A repeated measures ANOVA with a Geisser-Greenhouse correction and Dunnett's multiple comparisons test was carried out to compare the mean of the matched groups. A p-value ≤ 0.05 was considered significant.

RESULTS

According to the manufacturer, the investigated SEC columns can be re-used up to five times, with a cleaning step in between. We tested the effect of re-using a qEV 70 nm Gen2 10 mL column (Izon Science) up to five times on EV yield,purity and miRNA quantity of the pooled SEC fractions. In Supplementary Figures 5A-E, the fold change in EV- (miRNA), ApoA-, ApoB- and protein concentration after consecutive column use compared to the first-time use are shown. Supplementary Table 3 indicates that while EV and EV-miRNA yield was not affected, the concentration of lipoproteins showed a 4-to-5-fold increase after the second column use. In addition, protein concentrations increased 2.5-fold after the second use. Thus, re-using SEC columns increased the concentration of lipoproteins and proteins, already after the second use, and reduced EV purity. EV-miRNA quantity was not affected after consequtive column use.

REFERENCES

1. Welsh JA, Van Der Pol E, Arkesteijn GJA, et al. MIFlowCyt-EV: a framework for standardized reporting of extracellular vesicle flow cytometry experiments. *J Extracell Vesicles.* 2020;9(1):1713526.
